# The critical roles of caveolin-1 in lung diseases

**DOI:** 10.3389/fphar.2024.1417834

**Published:** 2024-09-24

**Authors:** Jiarun Fan, Siping Zheng, Maoping Wang, Xiaoliang Yuan

**Affiliations:** Department of Respiratory Medicine, First Affiliated Hospital of Gannan Medical University, Ganzhou, China

**Keywords:** caveolin-1, pneumonia, asthma, COPD, ali, pulmonary hypertension, pulmonary fibrosis

## Abstract

Caveolin-1 (Cav-1), a structural and functional component in the caveolae, plays a critical role in transcytosis, endocytosis, and signal transduction. Cav-1 has been implicated in the mediation of cellular processes by interacting with a variety of signaling molecules. Cav-1 is widely expressed in the endothelial cells, smooth muscle cells, and fibroblasts in the various organs, including the lungs. The Cav-1-mediated internalization and regulation of signaling molecules participate in the physiological and pathological processes. Particularly, the MAPK, NF-κB, TGFβ/Smad, and eNOS/NO signaling pathways have been involved in the regulatory effects of Cav-1 in lung diseases. The important effects of Cav-1 on the lungs indicate that Cav-1 can be a potential target for the treatment of lung diseases. A Cav-1 scaffolding domain peptide CSP7 targeting Cav-1 has been developed. In this article, we mainly discuss the structure of Cav-1 and its critical roles in lung diseases, such as pneumonia, acute lung injury (ALI), asthma, chronic obstructive pulmonary disease (COPD), pulmonary hypertension, pulmonary fibrosis, and lung cancer.

## 1 Introduction

Caveolae, a subset of lipid rafts with 50–100 nm in diameter, are the flask-shaped invaginations in the cell plasma membrane. There are three caveolin proteins, including caveolin-1 (Cav-1), Cav-2, and Cav-3. The genes *Cav-1* and *Cav-2* are located at chromosome 7q31.1, and *Cav-3* is located at 3q25. The three caveolins share significant homology. The amino acid sequence of Cav-2 has approximately 38% of identity and 58% of similarity to that of Cav-1. Cav-3 has a higher homology with approximately 65% of identity and 85% of similarity (Williams and Lisanti, 2004). Cav-1 and Cav-2 are ubiquitously co-expressed in many mammalian cells, whereas Cav-3 can be found in cardiac and skeletal muscle cells. Cav-2 is generally found associated with Cav-1 and serves as an accessory protein for correct caveola formation. Caveolins are the structural and functional components in the caveolae and play an important role in physiological and pathological events. Caveolins have been associated with transcytosis, endocytosis, and signal transduction (Anderson, 1998). Cavins are caveolae-associated proteins that include cavins 1–4 (Hill et al., 2008; McMahon et al., 2009).

Caveolae formation requires caveolins, cavins, EH-domain containing protein 2 (EHD2), pacsin2 (syndapin II), and lipids (cholesterol and sphingolipids). Cavin-1 is essential for the generation of the flask-shaped structure in caveolae. Caveolae are enriched in cholesterol, which is bound by the Cav-1 scaffolding domain (Hubert et al., 2020). The specific binding of cavins to lipids may drive the membrane curvature (Lundmark et al., 2024). Cav-1 and cavin-1 are believed to form the caveolae coat machinery required to bend the membrane into the flask-shaped structure (Liu et al., 2008). Cavin-1 stabilizes Cav-1 by inhibiting its internalization and lysosomal degradation, indicating the critical role of cavin-1 in mediating Cav-1 expression and caveolae formation (Meng et al., 2015). The interaction between Cav-1 and cavin-3 is not required for caveolae biogenesis but for caveolae surface stability (Mohan et al., 2015). Caveolae emerge as the critical sensors at the plasma membrane, and the compartmentalization of certain signaling molecules within caveolae may significantly improve the efficiency of signaling transduction. The importance of caveolae has been highlighted by the association between caveolae dysfunction and human diseases (Parton and del Pozo, 2013). One study shows that Cav-1 regulates lipid transport, inflammatory responses, and has atherogenic effects, indicating the critical role of Cav-1 in atherosclerosis development (Shu and Jin, 2023). Cav-1 has been involved in the initiation and progression of cognitive decline (Tang et al., 2021). The roles of Cav-1 in the development of cancer, neurodegeneration, glaucoma, cardiovascular diseases, and infectious diseases have been discussed (Gokani and Bhatt, 2022).

The localization of Cav-1 can be caveolae-dependent and -independent (Bhowmick et al., 2023). The intracellular membranes for Cav-1 localization include the endoplasmic reticulum (ER) and late endosomal/lysosomal membranes. Therefore, Cav-1 might exhibit multiple biological activities independent of caveolae (Enyong et al., 2022). Caveolae are crucial factors for sensing and transducing mechanical forces. However, it is reported that Cav-1 exhibits deformability and mechanoprotection independent of caveolae by stabilizing non-caveolar invaginations-dolines, which are capable of responding to low-medium mechanical forces and impacting downstream mechanotransduction (Lolo et al., 2023). Non-caveolar Cav-1 has been associated with increased expression of VEGFA in lymphatic endothelial cells (Nassar et al., 2015). The expression of cavin-1 is absent in PC3 and LNCaP cells but abundant in DU145 cells. Cavin-1 deficiency leads to the abundance of Cav-1 and the absence of caveolae. Non-caveolar Cav-1 can be detected on the flat plasma membrane (Nassar et al., 2015). Cav-1 has been shown to mediate cellular processes, such as cell proliferation, differentiation, migration, and survival (Dalton et al., 2023). It is suggested that lipid rafts/caveolae play a role in maintaining the self-renewal of embryonic stem (ES) cells. Treatment with Cav-1 siRNA and methyl-β-cyclodextrin induces the downregulation of octamer-binding protein 4 (Oct4), SRY-box transcription factor 2 (Sox2), Forkhead box D3 (FoxD3), RNA exonuclease 1 homolog (Rex1), G1/S-specific cyclin-D1 (cyclin D1), and cyclin E and the reduction of the proliferation index in ES cells (Lee et al., 2010). Dysregulation of Cav-1 may result in the occurrence of pathophysiological processes and the development of diseases. Enhanced expression of Cav-1 has been associated with high levels of glycolysis and metastasis in melanoma and breast cancer cells (Díaz-Valdivia et al., 2022). Decreased expression of Cav-1 is observed in postmenopausal osteoporosis. Irisin, a myokine, has been reported to protect against osteoporosis development by up-regulating the expression of Cav-1 (Tao et al., 2024).

The incidence of lung diseases has increased in recent years due to altered lifestyles and environmental pollution, and lung diseases have become a major cause of death and disability worldwide (GBD Chronic Respiratory Disease Collaborators, 2020). The most common lung diseases include pneumonia, asthma, chronic obstructive pulmonary disease (COPD), acute lung injury (ALI), pulmonary hypertension, pulmonary fibrosis (PF), and lung cancer. Notably, COPD, asthma, and lung cancer rank among the top 10 causes of death (Levine and Marciniuk, 2022). Pneumonia, due to its high incidence and susceptibility to complications, poses a significant health concern. Particularly, the global pandemic of coronavirus pneumonia (COVID-19) has had a profound impact on human life and health since 2019 (Ocampo et al., 2024). Accumulating evidence suggests that precise and targeted medical interventions are important, necessitating a thorough understanding of the pathogenesis of lung diseases. Caveolae and Cav-1 are widely distributed in alveolar epithelium, airway, pulmonary artery smooth muscle, and fibroblasts, indicating their potential role in the pathophysiological alterations associated with lung diseases. For instance, caveolae and Cav-1 have been involved in the mediation of agonist-induced [Ca^2+^]_i_ signaling and the hyperresponsiveness of airway smooth muscle (ASM) (Sathish et al., 2011). In this article, we primarily focus on discussing the critical roles of Cav-1 in lung diseases.

## 2 Methods of literature research

The related articles published up to April 2024 were collected from the databases, such as Pubmed Web of Science, Google Scholar, ScienceDirect, and SpringerLink. The searched keywords included caveolin-1, inflammation, pneumonia, asthma, COPD, ALI, pulmonary hypertension, PF, and lung cancer.

## 3 Cav-1: structure and its roles in inflammation

### 3.1 The structure of Cav-1

**FIGURE 1:**
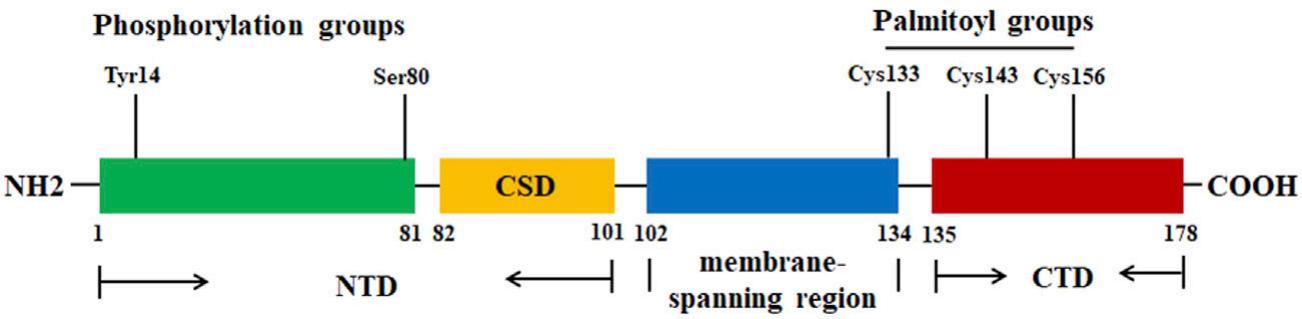
The protein structure of Cav-1. The Cav-1 protein has three regions: NTD (residues 1–101), membrane spanning region (residues 102–134), and CTD (residues 135–178). CSD (residue 82–101) is the region for Cav-1 to interact with various signaling molecules. The residues Tyr14 and Ser80 are the two phosphorylation sites. The residues Cys133, Cys143, and Cys156 can be palmitoylated.

Cav-1, a 178-amino acid protein, has three distinctive regions: a hydrophilic N-terminal domain (NTD, residues 1–101), a membrane-spanning region (residues 102–134), and a hydrophilic C-terminal domain (CTD, residues 135–178) (Ohi and Kenworthy, 2022) (Figure 1). In the NTD, there is an invariant structure FEDVIAEP stretch, which has been known as the caveolin signature motif (residues 68–75) (Scherer et al., 1996). There are only three cysteine (Cys) residues (Cys133, Cys143, and Cys156) that are located at the CTD, and the three Cys residues can be palmitoylated (S-acylation) (Figure 1), indicating that the CTD is linking to the membrane (Dietzen et al., 1995). Cav-1 has a caveolin scaffolding domain (CSD, residue 82–101), which may interact with and mediate the activity of various signaling molecules. For example, Cav-1 can interact with cholesterol due to the recognition consensus VTKYWFYR (residues 94–101) and mediate the cholesterol levels in mitochondria. It is reported that Cav-1 has two isoforms: Cav-1α (24 kDa) and Cav-1β (21 kDa). Both isoforms have a complete CTD, indicating that both can be localized within the caveolae. However, Cav-1β lacks an N-terminal-specific protein sequences (residues 1–21). This might be explained by the two alternate translational start sites (Met1 and Met32) (Scherer et al., 1995).

Cav-1 is synthesized in the ER. Cav-1 has been reported to interact with itself to form a high-order oligomeric complex (approximately 350 kDa–400 kDa) comprised of 14–16 monomers. The presence of an ER export signal in the NTD promotes the release of Cav-1, which can be assembled into 8S complexes that accumulate in the ER exit sites. Next, Cav-1 can be further assembled into aggregates with higher molecular weight of 130–150 monomers in the Golgi (Hayer et al., 2010a). The accumulation of Cav-1 in the Golgi may lead to the loss of diffusional mobility, the occurrence of conformational changes, the association with cholesterol, and finally the assembly of 70S complexes (Hayer et al., 2010a). It is important to note that the oligomerization of caveolin might be the driving force for the assembly of caveolae (Fra et al., 1995). The NTD has been associated with the homooligomerization activity, which is independent of disulfide bond formation. Mutations in residues 1–21, 1–61, 71–101, 76–101, or 81–101 block the formation of high-order oligomers. However, it is interesting to find that GST-Cav-1α fusion and GST-Cav-1β fusion can co-migrate as high molecular mass oligomers in velocity gradients (Sargiacomo et al., 1995). Consistently, it is reported that the juxtamembrane region of Cav-1 is associated with the oligomerization activity (Couet et al., 1997a).

In addition, two phosphorylation sites Tyr14 and Ser80 (Figure 1) locate at the regulatory amino-terminal tail. Src-mediated phosphorylation of Cav-1 Tyr14 is associated with the spatial organization of Cav-1 within the oligomer, mediating the internalization (Zimnicka et al., 2016). Cav-1 Ser80 phosphorylation may affect its cellular localization within the ER, stimulate its secretory pathway, and contribute to its tumor-suppressive activity (Schlegel et al., 2001; Díaz et al., 2020). One study reports that binding of caveolin with heterotrimeric G-proteins and Src may lead to the suppression of their enzymatic activity and inhibition of signaling transduction. It has been shown that the interaction between CSD and caveolin-binding molecules (CBMs) may induce the inactivation of the signaling molecules. For example, Cav-1 may act as an inhibitor of kinases, such as epidermal growth factor receptor (EGFR) and protein kinase C (PKC) (Couet et al., 1997b). However, the binding activity of Cav-1 may be independent of CSD-CBM interactions. Some molecules, such as steroid hormone receptors, tyrosine kinase receptors, G-protein-couple receptors, and G-proteins, do not have the CBMs and can interact with Cav-1 (Bhowmick et al., 2023). When the levels of Cav-1 are abnormally enhanced, the accumulated Cav-1 can be ubiquitinated and subsequently degraded within endolysosomes (Hayer et al., 2010b). One study reports that LPS treatment promotes the binding of E3 ubiquitin ligase ZNRF1 to Cav-1 and induces its degradation. Interestingly, activation of the ZNRF1/Cav-1 signaling may lead to increased production of pro-inflammatory cytokines (Lee et al., 2017).

Cav-1 can physically interact with various proteins via CSD and inhibit their activities (Van Krieken and Krepinsky, 2017). Heme oxygenase-1 (HO-1) catalyzes the O_2_-dependent degradation of heme to carbon monoxide (CO), free ferrous iron, and biliverdin IXα, playing an essential role in counteracting oxidative stress. HO-1 can bind to Cav-1, which exhibits an inhibitory activity against the enzymatic activity of HO-1. In addition, the core binding site of Cav-1 is available for HO-1 (Taira et al., 2011). One study shows that Cav-1 with deletion of the 101^th^ residue (Δ101CSD peptide) can interact with HO-1 and disrupt the interaction between HO-1 and wild-type (WT) Cav-1. However, Δ101CSD peptide cannot enhance HO-1 activity but increase HO-1 mRNA expression. In addition, Δ101CSD peptide impairs the inhibitory activity of WT Cav-1 against the MAPK signaling and inflammatory responses (Jin et al., 2018). Structure-functional study shows that Phe92 is an essential residue for the inhibitory activity of Cav-1 against endothelial nitric oxide synthase (eNOS), and residue 90–99 in Cav-1 is the binding site for eNOS (Trane et al., 2014). It is reported that F92A-Cav-1 can abrogate the inhibitory effects of Cav-1 against eNOS and promote NO synthesis by disrupting their interaction (Bernatchez et al., 2011). Mechanically, F92A-Cav-1 increases the phosphorylation of AKT, which is one of the most prominent activators of eNOS. However, F92A-Cav-1 does not affect endogenous Cav-1 oligomerization and Cav-1 and eNOS distribution (Trane et al., 2015).

One study reports that CSD (residue 82–101) is split into three slightly overlapping shorter peptides (82–89, 88–95, and 94–101). Interestingly, all three peptides exhibit beneficial effects on bleomycin-treated mice. Both 82–89 and 88–95 peptides show inhibitory activity against fibrosis and microvascular leakage, improving ventricular functions. However, the 94–101 peptide merely decreases Collagen I expression (Reese et al., 2022). It has been reported that the GC-rich sequences (−372/−222 and −150/−91) in the mouse Cav-1 promoter are oxidative stress-responsive elements. Sp1 has been known to interact with the GC-rich boxes. Under oxidative stress, Sp1 can positively increase the promoter activity of Cav-1. Hydrogen peroxide treatment activates p38 MAPK, which upregulates Sp1-mediated Cav-1 expression in NIH 3T3 cells (Dasari et al., 2006).

### 3.2 The functional roles of Cav-1 in inflammation

**FIGURE 2:**
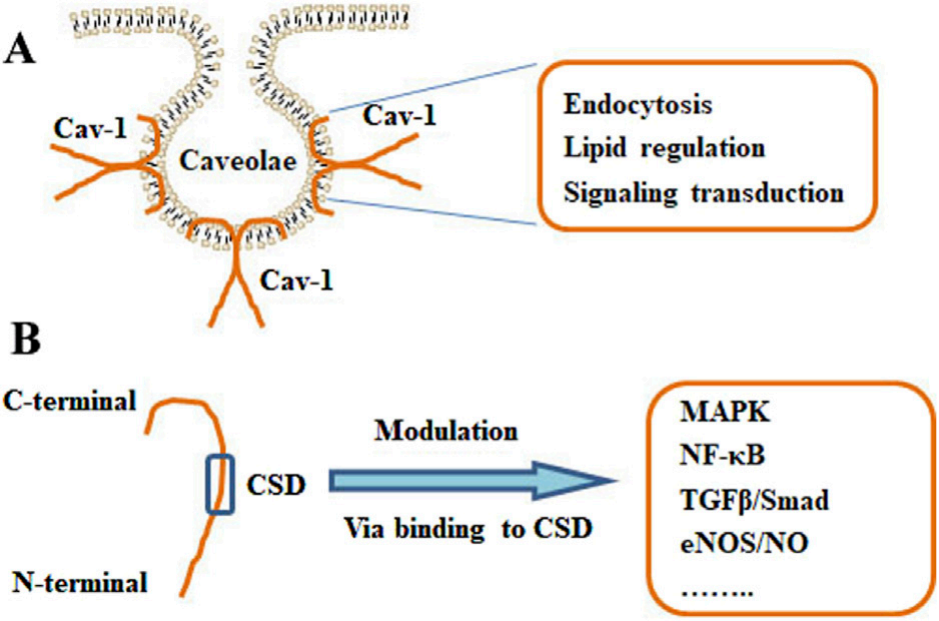
Structure and functions of Cav-1. **(A)** The localization of Cav-1 in caveolae. The primary actions of Cav-1 include endocytosis, lipid regulation, and signaling transduction. **(B)** The monomer of Cav-1 and the Cav-1 scaffolding domain (CSD) were shown. Cav-1 can modulate various pathways, such as MAPK, NF-κB, TGFβ/Smad, and eNOS/NO signaling, via binding to the CSD.

Cav-1 has been associated with multiple cellular processes, such as immune responses, endocytosis, membrane trafficking, cellular signaling, and cancer progression (Shi et al., 2020) (Figure 2). Cholesterol is essential for forming and maintaining caveolae. Cav-1 can interact with free cholesterol and mediate cholesterol transport through a lipoprotein chaperone complex, which consists of Cav-1, cyclophilin A, cyclophilin 40, and HSP56. Cav-1 facilitates the uptake of cholesterol via this chaperone complex, and Cav-1 has been implicated in the transport of newly synthesized cholesterol from the ER to the plasma membrane (Lin et al., 2009). Caveolae present as a platforms for lipid-based signaling transduction through compartmentalizing and concentrating signaling molecules. Interestingly, Cav-1 exhibits a negative mediator of various cellular signaling pathways (Dessy et al., 2010). Cav-1 may recruit lipids and proteins to caveolae and negatively mediate the transduction of signaling pathways, which include tyrosine kinases and receptor tyrosine kinases, Gα subunits, GTPases, c-Src, H-Ras, eNOS, and components of the MAPK pathway (Feng et al., 2013). The interaction between Cav-1 and different lipid bodies (lipid rafts, lipid droplets, cholesterols, sphingolipids, and fatty acids) has been reviewed (Bhowmick et al., 2023).

It has been reported that Cav-1 plays a role in inflammatory responses. Cav-1 can decrease the production and release of various inflammatory cytokines, such as interleukin 1β (IL-1β), IL-2, IL-4, IL-12, tumor necrosis factor α (TNFα), granulocyte and macrophage colony-stimulating factor (GM-CSF), and regulated upon activation, normal T-cell expressed and secreted (RANTES) (Qin et al., 2016). Mechanically, caveolae provide an ideal platform for signaling transduction. It has been demonstrated that many different types of receptors or signaling molecules are associating with caveolin proteins (Pike, 2005; Chidlow and Sessa, 2010). Many receptors, such as MAPK pathway components, receptor tyrosine kinases (RTKs), and GTPase have been sequestered within caveolae by interacting with Cav-1 (Anderson et al., 1992; Schnitzer et al., 1996). Cav-1 can enhance the phosphorylation of p38 but suppress that of c-Jun N-terminal kinase (JNK), Akt, and ERK1/2. SB203580, an inhibitor of p38 MAPK, can abolish the effects of Cav-1 on lipopolysaccharide (LPS)-induced inflammatory responses (Wang et al., 2006). Cav-1 silence may block IL-1β-induced activation of p38 MPAK, reducing tube formation and migration of endothelial cells (ECs) (Jagielska et al., 2012). COX-2, a mediator of inflammation, is localized in the ER, nuclear envelope, cavelae-like structures. The interacting of COX-2 and Cav-1 in the ER may induce the degradation of COX-2. Knockdown of Cav-1 may enhance the protein levels of COX-2 (Chen S. F. et al., 2010). The eNOS/NO signaling pathway also plays a critical role in inflammatory responses. The associating of Cav-1 with eNOS can inhibit the release of eNOS and the synthesis of NO (Bucci et al., 2000).

Cav-1 can interact with toll-like receptors (TLRs) to mediate phagocytosis and cell activation (Sivanantham et al., 2023). LPS can recognize and activate TLR4, leading to the recruitment of the adaptor MyD88, IL-1R-associated kinase 1 (IRAK1), IRAK4, and TRAF6 and the activation of TAK1. Consequently, TAK1 activation can induce the stimulation of NF-κB and increase the production of pro-inflammatory cytokines, such as IL-1, IL-6, IL-12, TNFα, and macrophage inflammatory protein (MIP). It is reported that Cav-1 interacts with TLR4 and inhibits the assembly of TLR4 complex with MyD88. Interestingly, the binding motif for Cav-1 in murine TLR4 (^739^FIQSRWCIF^747^) has been identified. W744A mutation in TLR4 may abolish the interaction of Cav-1 with TLR4 (Wang et al., 2009). Another study shows that TLR4-mediated recruitment of MyD88 may induce Cav-1 Tyr14 phosphorylation, IκB degradation, and translocation of NF-κB to the nucleus (Jiao et al., 2013). Recently, it is reported that oxidized low-density lipoproteins (oxLDLs) may disrupt the eNOS/iNOS balance by mediating the HGMB1/TLR4/Cav-1 signaling, leading to the impairment of autophagic/apoptotic responses in vascular and nonvascular cells (Gliozzi et al., 2019). The discrepancy of the regulatory effects of Cav-1 on the TLR4 signaling might be associated with many factors, such as cell lines and micro-environment. Thus, more efforts are still needed.

Caveolae and caveolins are also expressed in macrophages. In LPS-treated murine alveolar and peritoneal macrophages, Cav-1 can decrease the production of TNFα and IL-6 and increase the generation of IL-10 (Wang et al., 2006). Cav-1 deletion has been associated with enhanced inflammatory responses, decreased phagocytic activity of macrophages, and increased superoxide release (Yuan et al., 2011). Cav-1 expression and intracellular cholesterol levels are mutually mediated to maintain cholesterol homeostasis. In the pathological processes of atherosclerosis, macrophages derived from monocytes are transformed into lipid-loaded cells via uptake of lipoproteins, especially ox-LDL. It is reported that Cav-1 interacts with scavenger receptor-BI (SR-BI), which regulates the transfer of lipid from lipoproteins into cells via selective cholesterol. Co-expression of Cav-1 and SR-BI can lead to increased uptake of selective cholesterol in macrophages (Matveev et al., 1999; Puddu et al., 2023). In the Cav-1-deleted mice, the levels of free cholesterol in peritoneal macrophages are decreased and the expression of ABCA1 in macrophages is also decreased. Cav-1 deficiency decreases the synthesis of free cholesterol (Lin et al., 2007; Qin et al., 2016). In addition, macrophages derived from Cav-1-deleted mice show severe LPS-induced inflammatory responses (Medina et al., 2006). The co-localization of TLR4 with Cav-1 in caveolae of peritoneal macrophages indicates a connection between Cav-1-regulated cellular cholesterol efflux and the inflammatory responses (Wang et al., 2009). PPARγ and RXR may promote the expression of Cav-1, SR-BI, and ABCA1. Activation of PPARγ and/or RXR leads to upregulation of Cav-1 expression and downregulation of NF-κB, STAT, and AP-1 in macrophages (Luo et al., 2010).

Sterile inflammation is a process that reside cells sense pro-inflammatory signals, release extracellular mediators, and then recruit circulating immune cells that trigger and escalate the inflammatory responses. Various cell types, including mesothelial cells, endothelial cells, and immune cells (such as macrophages) may promote the initiation and progression of sterile inflammation. However, the exact mechanisms are not fully understood (Su et al., 2024). Endosomal TLRs recognize self-derived nucleic acids via damage-associated molecular patterns (DAMPs) in cases of apoptosis or cellular damage, triggering sterile inflammation. TLR-3, -7, -8, and -9 are located inside endosomes. TLR-3 recognizes double-stranded RNA, TLR-7 and TLR-8 detect single-stranded RNA, and TLR-9 identifies DNA (Zhou et al., 2020). However, LPS-induced endocytosis takes TLR4 to early endosome, where it recognizes endosomal LPS to activate MyD88-independent inflammatory responses (Kawai and Akira, 2010). Flotillin-1, a marker of membrane rafts, is essential for TLR signaling. Knockdown of flotillin-1 decreases TLR3-regulated inflammatory responses. Flotillin-1 and Cav-1 co-localize within the caveolae. The interaction between flotillin-1 and Cav-1 may facilitate the transport of TLR3-ligands to its intracellular receptor and activates inflammatory TLR3 signaling (Fork et al., 2014). Lumican is an ECM protein associated with collagens. Lumican can attach to Cav-1 and the TLR co-receptor CD14 and promote TLR4-but restrict TLR9-mediated inflammatory responses in macrophages (Maiti et al., 2021). During ischemia-reperfusion injury, endothelial DAMPs can decrease the expression of Cav-1 and eNOS and aggravate endothelial barrier disruption (Kumphune et al., 2024).

## 4 The roles of Cav-1 in lung diseases

### 4.1 Pneumonia and acute lung injury (ALI)

Pneumonia is a leading cause of global pediatric morbidity and mortality. Bacterial infections have been associated with activation of innate and adaptive immunity, regulation of antigen presentation, pathogen recognition, and phagocytosis. Cav-1 is required for pathogen invasion of host cells. However, Cav-1 negatively modulates inflammatory responses (Zaas et al., 2009). During infection, lung epithelial cells directly recognize the pathogen-associated molecular patterns via TLRs and trigger the innate immune responses for pathogen clearance (Grainge and Davies, 2013). ALI, a clinical syndrome of acute respiratory failure, is associated with acute lung inflammation, damaged alveolar-capillary barrier, airway edema, and abnormal gas exchange (Jones and Minshall, 2022). The potential mechanisms of ALI pathogenesis are still unclear. Cav-1 plays an important role in the pathogenesis of ALI. A bioinformatics study using GO, KEGG, and PPI analysis shows that the Cav-1/NF-κB signaling is one of the most effective targets for ALI prevention (Qu et al., 2022).

**FIGURE 3:**
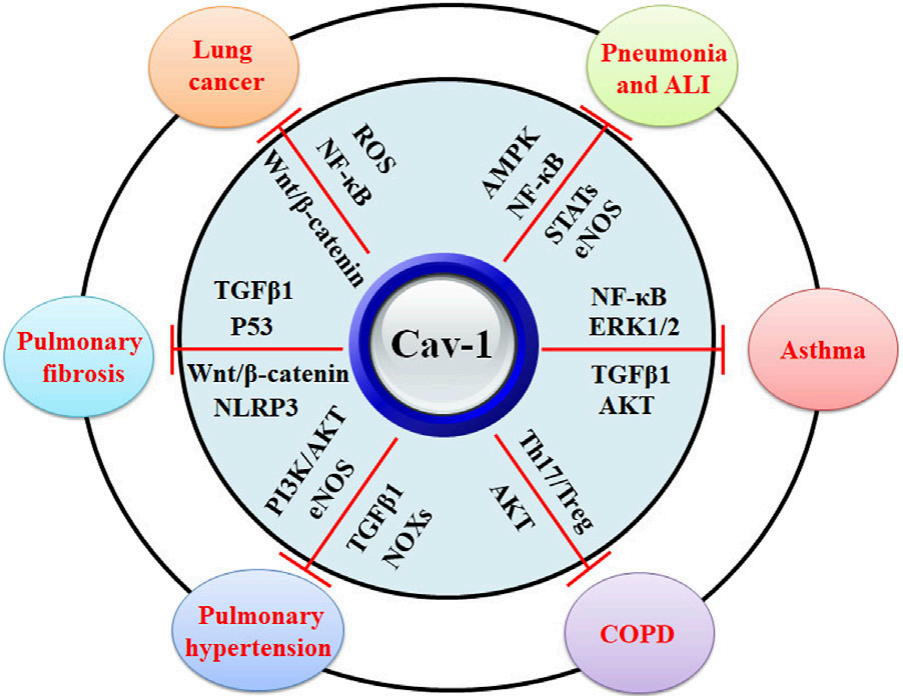
The biological functions of Cav-1 in protection against lung diseases. Cav-1 exhibits protective effects against pneumonia, ALI, asthma, COPD, pulmonary hypertension, pulmonary fibrosis, and lung cancers. The potential mechanisms might be associated with the inhibitory activity of Cav-1 against MAPK, NF-κB, STATs, eNOS, ERK1/2, TGFβ1, Th17/Treg disruption, NOXs, AKT, Wnt/β-catenin, and ROS.

In Cav-1 knockout mice, increased lung inflammatory responses and higher mortality rates are related to the enhanced STAT3/NF-κB signaling (Yuan et al., 2011) (Table 1) (Figure 3). It has been reported that Cav-1 can suppress LPS-induced inflammatory responses, microvascular barrier breakdown, and edema formation by inhibiting the NF-κB and eNOS signaling pathways (Garrean et al., 2006). One study shows that plasma albumin leakage, infiltration of immune cells, and levels of IL-6/IL-6R and p-TGFβ/p-Smad2/3 in LPS-treated Cav-1 knockout mice are significantly elevated. In contrast, the expression of BMPRII and the uncoupling of eNOS are reduced in LPS-treated ECs from Cav-1 knockout mice (Oliveira et al., 2017) (Table 1). TNFα contributes to various inflammatory conditions, including acute respiratory distress syndrome and COPD. TNFα plays a critical role in the activation of the NF-κB signaling, which can be mediated by the PI3K/AKT and p44/42 MAPK signaling pathways. TNFα acts as a ligand of TNF receptor (TNFR), which includes TNFR1 and TNFR2. In TNFR knockout mice, ozone-induced formation of the TNFR adaptor complex is attenuated, and activation of MAPK and NF-κB signaling pathways is suppressed (Cho et al., 2007). Ozone inhalation significantly induces TNFα expression and increases the PI3K/AKT and p44/42 MAPK signaling pathways, which can be negatively regulated by Cav-1 in alveolar macrophages (AMs) (Fakhrzadeh et al., 2008) (Table 1).

**TABLE 1:** The critical roles of Cav-1 in lung diseases.

Categories	Models	Biological actions	Ref.
Pneumonia	Cav-1^−/−^ mice	Cav-1 regulates the secretion of CCN1, which inhibits inflammatory responses	Moon et al. (2015)
	HULEC-5a cells	Cav-1 blocks PLY-induced barrier disruption and protects endothelial barrier integrity by promoting endocytosis of damaged membrane	Batori et al. (2022)
	Cav-1^−/−^ mice	Cav-1 protects against *K. pneumonia*-induced inflammatory responses and lung injury	Guo et al. (2012)
	MLE-12 cells	Cav-1 protects against *K. pneumonia* infection by mediating the STAT5/GSK-3β/β-catenin/AKT signaling pathway	
	HCECs	Cav-1 deficiency impairs FlgE-induced inflammation and ERK1/2 activation	Shen et al. (2017)
	COVID-19 patients	The expression of Cav-1 is decreased, and the expression of TGFβ1, α-SMA, and MMP-9 is increased	Vaz de Paula et al. (2021)
ALI	Cav-1^−/−^ mice	Cav-1 protects against ALI by negatively regulating the STAT3/NF-κB signaling	Yuan et al. (2011)
	Cav-1^−/−^ mice	Cav-1 suppresses LPS-induced the NF-κB and eNOS signaling pathways	Garrean et al. (2006)
	AMs	Cav-1 inhibits ozone-induced PI3K/AKT and p44/42 MAPK signaling pathways by decreasing the expression of TNFα	Fakhrzadeh et al. (2008)
	LPS-treated rats	Cav-1 is involved in the inhibitory effects of Sirt1 against ALI by decreasing the expression of STAT3, TLR4, TNFα, and IL-6	Tong et al. (2023)
	Cav-1^−/−^ mice	Cav-1 deficiency decreases BMPR2 expression and increases the TGFβ/Smad2/3 and IL-6/IL-6R signaling pathways	Oliveira et al. (2017)
	I/R rats	Cav-1 mediates the protective effects of Dexm against I/R-induced ALI by decreasing the p38 MAPK and NF-κB signaling pathways	Xu et al. (2021)
	HLMVECs	Cav-1 inhibits LLO-induced ALI by interacting with eNOS.	Chen et al. (2014b)
Asthma	Mice	CSD peptide abrogates hyperoxia-induced airway hyperresponsiveness and remodeling	Vogel et al. (2019)
	ASMCs	Cav-1 mediates the inhibitory effects of RXM on VEGF responses	Pei et al. (2016)
	ASMCs	Cav-1 is involved in the regulation of RXM in TGFβ1-induced proliferation	Dai et al. (2014)
	Spry2^−/−^ mice	Cav-1 interacts with CSK, decreases CSK/LCK interaction, and attenuates the expression of Spry2, which promotes T-cell-driven asthma	Sripada et al. (2021)
	PBECs	Cav-1 negatively regulates VEGF-mediated MUC5AC.	Kim et al. (2020)
	OVA-treated mice	Cav-1 negatively regulates the expression of GATA-6, which promotes the TLR2/MyD88 and NF-κB pathway	Fang et al. (2016)
COPD	PBMCs	Cav-1 contributes to Th17/Treg imbalance by regulating Hsp70 expression	Zhang et al. (2023a)
	AECs	CSP7 decreases mucus hypersecretion	Das et al. (2023)
	16HBE cells	Cav-1 promotes the expression of MUC5AC, p-EGFR, and p-AKT.	Yu et al. (2015)
	IL-1β-treated chondrocytes	Cav-1 is involved in IL-1β-mediated inflammatory responses and cell apoptosis by regulating the p38 MAPK signaling pathway	Zhou et al. (2022)
Pulmonary hypertension	Cav-1^−/−^ mice	Cav-1 deficiency is associated with an increase in artery stiffness	Moreno et al. (2021)
	Cav-1^−/−^ mice	Cav-1 deficiency induces eNOS hyper-activation	Zhao et al. (2009)
	PMVECs	Cav-1 deficiency enforces the protective effects of pravastatin against LPS-induced inflammatory responses and apoptosis	Ren et al. (2021)
	PAECs	Cav-1 deficiency induces the JAK/STAT and PI3K/AKT signaling pathways and activation of type I inflammatory responses	Gairhe et al. (2021)
	Cav-1^−/−^ mice	Cav-1 deficiency decreases BMPR2 localization at the plasma membrane and activation of Smad1/5/9 signaling	Tomita et al. (2024)
	Hypoxia-treated PASMCs	Cav-1 promotes PPARγ activation, decreases the expression of the proliferative mediators, and increases the expression of the apoptosis-related factors	Yang et al. (2018)
PF	Bleomycin-treated rats	Decreased Cav-1 promotes the pathogenesis of PF by increasing the TGFβ signaling	Xing et al. (2017)
	Fibroblasts	Cav-1 mediates the anti-fibrosis activity of CRTH2, which promotes the degradation of collagen	Zuo et al. (2021)
	Sftpc-mTOR^SL1+IT^ mice	Decreased Cav-1 expression is associated with increased activity of the mTOR/ANGPTL4 signaling induced by bleomycin	Saito et al. (2020)
	Bleomycin-treated mice	Cav-1 is a target of miR-199a-5p, which is a product of DNM3OS and promotes the profibrotic effect of TGFβ	Savary et al. (2019)
	Cav-1^−/−^ mice	Cav-1 inhibits silica-induced infiltration of inflammatory cells and secretion of inflammatory factors by suppressing the NF-κB signaling	He et al. (2022)
Lung cancer	Cav-1^−/−^ mice	Cav-1 deficiency is associated with NOX-derived ROS production and activation of the NF-κB signaling	Chen et al. (2014a)
	Cav-1^−/−^ mice	Cav-1 deficiency promotes the ubiquitination and proteasomal degradation of ATP7A, which regulates the expression of SOD3	Sudhahar et al. (2020)
	H460 cells	Cav-1 interacts with Oct4, inhibiting NO-induced stemness of lung cancer cells	Maiuthed et al. (2018)
	A549 cells	Cav-1 is essential for hnRNPA1-loaded sEV-miRNAs, which facilitate tumor proliferation and migration	Li et al. (2021)
	H23 cells	Cav-1 promotes SSH20 uptake and its cytotoxicity	Robb et al. (2021)
	H292, H460 cells	Cav-1 O-glcNAcylation induced by TRPM7 promotes the metastasis of lung cancer cells	Luanpitpong et al. (2020)
	H460 cells	Cav-1 inhibits the migration and invasion by suppressing the Wnt/β-catenin signaling	Song et al. (2012)
	H358-IRR and A549-IRR	Cav-1 promotes autophagy and enhances IR-resistant cell survival by increasing IRGM expression	Chen et al. (2021)

*Streptococcus pneumonia* has become a major cause of community-acquired pneumonia. Although effective antibiotics have been advanced, pneumonia-associated morbidity and mortality have increased worldwide (Nishimoto et al., 2020). Pneumolysin (PLY), a cholesterol-dependent cytolysin (CDC), is a bacterial pore-forming toxin and primary virulence factor, affecting the plasma membrane homeostasis in the endothelial cells and being involved in all stages of pneumococcal diseases. It has been reported that Cav-1 and CSD peptide can protect the endothelial barrier from the disruption induced by PLY by promoting the endocytosis of damaged membrane (Batori et al., 2022). *Klebsiella pneumoniae* is the 3^rd^ most commonly separated microorganism from patients with bacterial sepsis. In Cav-1 knockout mice, the infection of *K. pneumoniae* significantly decreases survival rate and increases pro-inflammatory cytokine production. Cav-1 exhibits protective effects against *K. pneumoniae* infection by mediating the signal transducer and activator of transcription 5 (STAT5)/GSK-3β/β-catenin/AKT signaling pathway (Guo et al., 2012) (Table 1).

*Pseudomonas aeruginosa* (*P. aeruginosa*) infection increases the production of pro-inflammatory cytokines in alveolar macrophages. In Cav-1 knockout mice, the sensitivity to *P. aeruginosa* increases, the mortality rate is enhanced, and the inflammatory responses are elevated. These indicate the important contribution of Cav-1 to the innate host immunity to *P. aeruginosa* (Gadjeva et al., 2010). Cav-1, a common component of extracellular vesicles (EVs), is exclusively expressed in the miRNA-rich EVs, which are derived from lung epithelium. The miRNA-rich EVs have been associated with *P. aeruginosa*-induced inflammasome activation, neutrophil recruitment, and M1-macrophage polarization (Lee et al., 2019). Flagellar hook protein E (FlgE) in *P. aeruginosa* has been reported to stimulate human corneal epithelial cells (HCECs), enhance the production of pro-inflammatory cytokines, and promote the Th2-biased humoral responses to ovalbumin*.* However, the knockdown of Cav-1 abrogates FlgE-induced inflammation and extracellular regulated protein kinase1/2 (ERK1/2) activation (Shen et al., 2017) (Table 1). Alpha-toxin (Hla), a major virulence factor of *staphylococcus aureus*, plays a critical role in pneumonia. Hla can interact with the cell surface of eukaryotic host cells and form heptameric transmembrane pores. Cav-1 acts as a pore-stabilizing factor and facilitates the binding of Hla to its receptor α5β1 integrin. However, both Cav-1 and α5β1 integrin are not associated with toxin sensitivity (Möller et al., 2020).

Gram-positive infection-induced ALI is characterized by impaired endothelial cell (EC) barrier integrity and extensive permeability, which is associated with the excretion of cholesterol-dependent cytolysins, such as PLY and listeriolysin (LLO). It has been reported that LLO treatment can induce Ca^2+^-activated PKCα and stimulate the dissociation of Cav-1 and Hsp90 from eNOS, uncoupling eNOS and promoting NO synthesis. However, Cav-1 peptide may block the effects of LLO-induced eNOS signaling in human lung microvascular endothelial cells (HLMVECs) (Chen et al., 2014b) (Table 1). However, one study reports that the upregulated expression of Cav-1 in polymorphonuclear neutrophils (PMNs) is associated with PMN activation, adhesion, and migration, stimulating lung inflammation and vascular injury (Hu et al., 2008). This discrepancy might be associated with the different cell lines and microenvironment.

Cav-1 has been associated with the internalization of some viruses, such as the BK virus (Moriyama et al., 2007) and Simian virus-40 (Pelkmans et al., 2001). It has been reported that there are several caveolin-binding sites in coronavirus (Padhan et al., 2007), supporting the relationship between SARS-CoV-2 and lung injury. It has been reported that some coronavirus family members can cause acute alveolar damage by stimulating the Cav-1 signaling. However, they do not enter the lung cells in a caveolin-dependent manner (Buckley et al., 1998). During viral infection, the lung epithelial cell injury induces an accumulation of TGFβ1, which can be negatively mediated by Cav-1. In the lung samples from COVID-19-infected patients, the expression of Cav-1 is downregulated, and the transforming growth factor β1 (TGFβ1) signaling pathway is enhanced (Vaz de Paula et al., 2021) (Table 1).

During lung injury, pulmonary surfactant protein C (SP-C) expression is decreased in type II alveolar epithelial cells (AECs). Decreased expression of SP-C contributes to cell apoptosis by enhancing the expression of p53 and activating the Src/Cav-1 signaling in AECs (Puthusseri et al., 2017). Sirt1 exhibits protective activity against LPS-induced ALI by inhibiting inflammatory responses. The Sirt1 agonist SRT1720 can upregulate Cav-1 expression and downregulate STAT3, TLR4, TNFα, and IL-6 expression in rats (Tong et al., 2023) (Table 1). Mechanical ventilation can induce ventilator-induced lung injury. Ropivacaine, an amide-linked local anesthetic, exhibits anti-inflammatory effects in ALI. It is reported that ropivacaine may decrease pulmonary edema, leukocyte infiltration, and vascular hyperpermeability by inhibiting the Src/Cav-1 signaling (Piegeler et al., 2014). The CpG motif in bacterial DNA has been associated with pathogen-induced inflammation. It is reported that the synthetic CpG oligonucleotide (ODN) can promote the secretion of CCN1, which stimulates host immune responses via mitogen-activated protein kinase (MAPK) and nuclear factor kappa-B (NF-κB) signaling pathways in epithelial cells (Dalpke et al., 2002) (Figure 3). Mechanically, CpG ODN induces ER stress, enhances the phosphorylation of Src and Cav-1, and increases the secretion of CCN1 by modulating the interaction of p-Cav-1 and the CCN1 IGFbp domain (Moon et al., 2015) (Table 1).

Oxidative stress promotes the pathogenesis of sepsis-triggered ALI. Cav-1 and adiponectin are important modulators of oxidative stress. In Cav-1 and adiponectin double knockout mice, LPS dramatically triggers oxidative stress, nitrative stress, inflammatory responses, lung vascular permeability, and mouse mortality rate. Treatment with a superoxide scavenger MnTMPyP may rescue LPS-induced ALI. This indicates the critical role of Cav-1 and adiponectin in negatively mediating oxidative/nitrative stress and inflammation (Cai et al., 2014). Intestinal ischemic reperfusion (I/R) can induce ALI. Dexmedetomidine (Dexm), a highly selective alpha2-noradrenergic receptor (α2AR) agonist, can alleviate pulmonary inflammation after intestinal I/R by up-regulating Cav-1 expression and inhibiting the p38 MAPK and NF-κB signaling pathways (Xu et al., 2021) (Table 1). Dexm also exhibits protective effects by mediating the Cav-1/MAPK/NF-κB signaling pathway in ALI induced by LPS (Liu et al., 2019) or heatstroke (Geng et al., 2019).

### 4.2 Asthma

Asthma is associated with paroxysmal/persistent wheezing, dyspnea, and coughing, and these might be due to inflammation, hyperresponsiveness, and remodeling in the airway. Airway smooth muscle cells (ASMCs) are the main effector for airway remodeling, and the proliferation and hypertrophy of ASMCs may result in irreversible pathological alterations in the airway (Yuan et al., 2024). ASMCs contain caveolae that expression constituent protein Cav-1 and Cav-2. It has been reported that the expression of Cav-1 in endobronchial biopsies of asthmatic patients is decreased (Bains et al., 2012). Decreased Cav-1 expression has been involved in airway diseases and contributed to airway hyperresponsiveness and remodeling. In hyperoxia-exposed animals, downregulated Cav-1 expression in ASM has been found, and intraperitoneal injection of CSD peptide can ameliorate hyperoxia-induced pathophysiological alterations (Vogel et al., 2019) (Table 1). One study shows that Cav-1-knockout mice have significantly high proliferation marker expression and airway hyperresponsiveness and remodeling (Aravamudan et al., 2012).

Cav-1 acts as a negative regulator of cellular signaling, such as the ERK1/2 signaling (Mao et al., 2022). Activation of the ERK1/2 signaling mediates the secretion, proliferation, and hyperresponsiveness of ASMCs. Inhibition of the ERK1/2 signaling can be the therapeutic strategy for asthma treatment. Roxithromycin (RXM) has been reported to inhibit vascular endothelial growth factor (VEGF)-induced SMC proliferation by up-regulating Cav-1 expression and inhibiting the ERK1/2 signaling (Pei et al., 2016). TGFβ1 secretion is significantly enhanced in asthmatic ASMCs, and it is positively correlated with ASMC proliferation. However, RXM inhibits TGFβ1-induced ASMC proliferation by up-regulating Cav-1 expression and down-regulating p-ERK1/2 and p-AKT expression (Dai et al., 2014) (Table 1) (Figure 3).

Vasoactive intestinal peptide (VIP), a gastrointestinal hormone, participates in various biological effects, including smooth muscle relaxation, modulation of immune response, and immune homeostasis maintenance (Gonzalez-Rey and Delgado, 2007). VIP suppresses airway remodeling *in vivo* and inhibits IL-13-induced ASMC proliferation by up-regulating Cav-1 expression and decreasing the phosphorylation of ERK1/2 (Wang et al., 2018). Sprouty2 (Spry2) is a well-known mediator of Ras/ERK signaling and a positive regulator of CD4^+^ T cell function and type II immunity. In *Spry2*-knockout mice, ERK1/2 activation, T-cell differentiation, and cytokine secretion are abrogated. Spry2 stimulates T-cell-driven asthma by increasing the levels of Th2 cytokines. Mechanically, Spry2 promotes ubiquitin proteasome-mediated degradation of Cav-1, which binds and enhances CSK activity. *Spry2* deficiency increases Cav-1/CSK association, enhances CSK/LCK interaction, abrogates ERK1/2 activation, and diminishes TCR-induced responses in CD4^+^ T cells (Sripada et al., 2021) (Table 1).

MUC5AC, a major mucin glycoprotein, is responsible for the viscoelastic property of mucus and can be hypersecreted in asthmatic subjects. VEGF expression is correlated with inflammation and airway blood vessel remodeling in asthma. VEGF can enhance the expression of MUC5AC by stimulating VEGFR2 (Kim et al., 2020). VEGFR2 and Cav-1 are co-localized on caveolae, and Cav-1 may negatively regulate the VEGFR2 signaling (Wu et al., 2013). Glucocorticoids (GCs), such as dexamethasone (Dex), are the first-line drugs for treating airway inflammation in asthmatic patients. It has been reported that Dex decreases VEGF-induced MUC5AC expression in PBECs by up-regulating Cav-1 expression and increasing the interaction between Cav-1 and VEGFR2 (Kim S. H. et al., 2019; Kim et al., 2020). Licochalcone A, a chalcone from licorice root, has been shown to suppress ASM cell proliferation by inhibiting the activation of VEGF, VEGFR2, and ERK1/2 and blocking the downregulation of Cav-1 expression (Kim et al., 2017).

However, another study shows that MUC5AC expression is mediated by NF-κB, which can be stimulated by intracellular Ca^2+^ signaling. Cav-1-containing lipid raft aggregation is involved in Ca^2+^ influx, NF-κB activation, and MUC5AC expression in bronchial epithelial cells (Xia et al., 2017). Cav-1 is an important mediator of IgE-dependent store-operated Ca^2+^ entry (SOCE) and promotes Orai1 expression. MS4A gene family is clustered around 11q12-13, a region associated with allergy and asthma susceptibility. MS4A4A has been reported to enhance PLCγ1 phosphorylation, SOCE, and degranulation by interacting with Cav-1 in human mast cells (Arthur et al., 2020). The pro-inflammatory cytokine TNFα can increase agonist-induced [Ca^2+^]_i_ responses in ASM. Knockdown of Cav-1 decreases the responses of [Ca^2+^]_i_ to histamine and blocks TNFα-triggered [Ca^2+^]_i_ responses in ASM (Sathish et al., 2014). In addition, the knockdown of Cav-1 decreases caveolar and Orai1 expression and blunts SOCE in TNFα-treated ASM (Sathish et al., 2012). Consistently, Cav-1 siRNA transfection blocks the amplitude and frequency of arachidonic acid (AA)-induced [Ca^2+^]_i_ oscillations in human ASM (Thompson et al., 2014). One study shows that the expression of Src and p-Cav-1 Tyr14 in toluene diisocyanate (TDI)-induced asthma mice is significantly increased. Inhibition of the Src/Cav-1 axis and RAGE expression can improve the redistribution of β-catenin from the membrane to cytosolic compartments, attenuating TDI-induced airway inflammation (Zhao et al., 2018). TDI exposure upregulates the expression of autotaxin (ATX) and its major product lysophosphatidic acid (LPA). Interestingly, Cav-1 is essential for the induction of ATX, but not IL-1β, by TDI (Broström et al., 2018).

It has been reported that Cav-knockout mice develop increased hyperresponsiveness and thickness of the subepithelial layers (Gabehart et al., 2013). Increased extracellular matrix (ECM) deposition contributes to airway remodeling. It is reported that treatment with CSD peptide may abrogate hyperoxia-induced ECM alterations by maintaining the balance between matrix metalloproteinases (MMPs) and their inhibitors (TIMPs) in human fetal ASMCs (Vogel et al., 2017). Upregulated expression of GATA-6 promotes airway inflammation and remodeling in a murine model of chronic asthma. Down-regulation of GATA-6 decreases ovalbumin (OVA)-induced inflammation, infiltration, mucus production, and MMPs (MMP-2 and MMP-9) and a disintegrin and metalloproteinase (ADAMTSs). However, the expression of Cav-1 is inversely related to the abundance of GATA-6 in mice with asthma (Fang et al., 2016) (Table 1). Consistently, Roxithromycin can decrease the thickness of the bronchial wall and bronchial smooth muscle cell layers and the phosphorylation of p42/p44 MAPK and increase the expression of Cav-1, suppressing airway remodeling in rats (Wu et al., 2015).

### 4.3 Chronic obstructive pulmonary disease (COPD)

Chronic obstructive pulmonary disease (COPD), the third leading cause of death, is characterized by airflow limitations due to chronic bronchitis and alveolar emphysema, while no effective treatments are available to reduce the mortality or inhibit the pathological progression due to a lack of full understanding the underlying mechanisms. The 3′-untranslated region single nucleotide polymorphisms (SNPs) of the Cav-1 gene loci rs8713 and rs1049337 have been reported to be related to a risk of pulmonary hypertension in patients with COPD (Li et al., 2019). Consistently, the expression of Cav-1 in medial smooth muscle cells of pulmonary arteries in patients with COPD has been reported to be correlated with pulmonary hypertension (Huber et al., 2009). Multiple immune cells, such as CD4^+^ and CD8^+^ T lymphocytes, and inflammatory cytokines have been implicated in the immunopathogenesis of COPD. The roles of T helper 17 (Th17) cells and regulatory T (Treg) cells in the development of COPD have been reviewed (Ma et al., 2023). Hsp70 plays a role in inflammation and innate immunity response and contributes to the conversion of Treg cells into Th17 cells (Guo et al., 2018). In COPD patients, the expression of Cav-1 is decreased. Cav-1 disrupts the balance between Th17 and Treg cells by mediating Hsp70 expression (Figure 3). In addition, Cav-1 overexpression may lead to an increase in Hsp70 and Th17 levels in peripheral blood mononuclear cells (PBMCs) (Zhang et al., 2023a) (Table 1).

Cigarette smoke (CS) exposure has become a risk factor for the pathogenesis of COPD. In CS-exposed airway epithelial cells (AECs) and type II alveolar epithelial (AT2) cells, the expression of Cav-1, p53, and plasminogen activator inhibitor-1 (PAI-1) is significantly upregulated. Treatment with a seven amino acid CSD peptide (CSP7) can decrease mucus hypersecretion in CS-exposed AECs (Das et al., 2023). CS extract (CSE) can enhance MUC5AC expression and increase EGFR and AKT phosphorylation levels in 16HBE cells. Cav-1 knockdown can abrogate CSE-induced up-regulation of MUC5AC, p-EGFR, and p-AKT expression (Yu et al., 2015) (Table 1). Autophagy-related factor LC3B has been associated with CS-induced lung epithelial cell death. Cav-1 deficiency can cause higher levels of autophagy and apoptosis in CS-treated mouse lungs (Chen Z. H. et al., 2010). Cav-1 deficiency in lung fibroblasts can suppress CS-induced premature senescence by inactivating the ATM/p53/PP2A-C signaling pathway (Volonte and Galbiati, 2009; Volonte et al., 2009). Treatment of bronchial chondrocytes with IL-1β can generate a stable COPD-related tracheobronchomalacia (TBM) cell model. The Tiao-bu-fei-shen (TBFS) formula, a traditional Chinese medicine, can significantly reduce inflammatory responses and cell apoptosis by down-regulating the Cav-1/p38 MAPK signaling pathway (Zhou et al., 2022) (Table 1).

### 4.4 Pulmonary hypertension

Pulmonary hypertension is associated with high mean pulmonary arterial pressure (more than 25 mmHg at rest) and pulmonary vascular resistance (more than 3 Wood units). Endothelial dysfunction, pulmonary vasoconstriction, and vascular remodeling are the main characteristics, and they may lead to increased pulmonary artery pressure, right ventricular hypertrophy, right heart failure, and premature death (Zaiman et al., 2005). Many signaling molecules, such as endothelial NO synthase (eNOS), VEGF receptor, prostacyclin receptors, bone morphogenetic proteins (BMPs), and TGFβ, have been implicated in the pathogenesis of pulmonary hypertension (Chettimada et al., 2015) (Figure 3). The expression of these molecules may be mediated by Cav-1. In addition, one study reports that Cav-1 deficiency is associated with an increase in collagen content and artery stiffness, promoting the development of pulmonary hypertension (Moreno et al., 2021) (Table 1). The critical roles of Cav-1 in the development of pulmonary hypertension have been comprehensively reviewed in recent years (Chettimada et al., 2015; Mathew, 2021).

Nitro oxide (NO), synthesized by eNOS, plays an important role in cardiovascular homeostasis. Genetic deletion of eNOS may lead to various cardiovascular phenotypes, such as elevated blood pressure, impaired angiogenesis, and abnormal vascular remodeling (Fernández-Hernando et al., 2006). It has been reported that the interaction between eNOS and caveolin-1 in caveolae leads to enzyme inhibition of eNOS. Upon stimulation, eNOS is translocated from the caveolae to the cytoplasm where it produces NO. *Chlamydia pneumoniae*, a Gram-negative bacterium, infects epithelial cells in the respiratory tract. *C. pneumoniae* can colocalize with eNOS, interfere with its trafficking from the Golgi apparatus to the plasma membrane, and inhibit NO synthesis in AECs (Mueller and Wolf, 2015). ECs synthesize low levels of NO, which maintains vessel homeostasis. Inflammation contributes to the dysfunction of ECs and affects the eNOS/NO signaling. The absence of Cav-1, hyper-activation of eNOS, and impairment of PKG activity may lead to vascular damage and remodeling and the development of pulmonary hypertension (Zhao et al., 2009) (Table 1).

Inflammation can disrupt the alveolar endothelial barrier and promote pulmonary microvascular permeability, leading to the development of ALI. Pravastatin, an inhibitor of HMG CoA reductase, exhibits anti-inflammatory activity. It is reported that pravastatin suppresses sepsis-induced inflammatory responses and apoptosis in LPS-treated pulmonary microvascular endothelial cells (PMVECs) by mediating the Cav-1/eNOS signaling pathway (Ren et al., 2021). The expression of STAT and PI3K/AKT is constitutively activated in PAECs. Cav-1 deficiency induces the JAK/STAT and PI3K/AKT signaling pathways and activation of type I inflammatory responses. Cav-1 silence also promotes AKT-induced phosphorylation of NOS3 Ser1177. Knockdown of NOS3 can abrogate the activation of STAT and AKT in PAECs (Gairhe et al., 2021) (Table 1).

BMP2 receptor (BMPR2) interacts with Cav-1 in the caveolae, and it is commonly mutated in pulmonary hypertension. Decreased BMPR2 signaling contributes to the hyper-activation of the TGFβ pathway, switching from the protective p-Smad1/5/8 signaling to the p-Smad2/3 pathway in pulmonary vascular cells (Erewele et al., 2022). In Cav-1 knockout mice, the localization of BMPR2 at the plasma membrane is attenuated, the phosphorylation of Smad1/5/9 is decreased, and the BMP/Smad signaling is inhibited. Cavin-1 competitively attenuates the interaction of Cav-1 with BMPR2 and inhibits the BMP/Smad signaling (Tomita et al., 2024). Consistently, Cav-1 depletion in ECs is also associated with pulmonary hypertension in hypoxia-treated rats. Cav-1 depletion reduces BMPR22 expression and increases the TGFβ/Smad2/3 signaling in the lung (Oliveira et al., 2019). In *BMPR2* ^*+/R899X*^ mice, the expression of Cav-1 is decreased, and the eNOS/NO signaling is increased in the lung. In addition, the pulmonary TGFβ levels are increased (Erewele et al., 2022). Cav-1 is negatively associated with the TGFβ/Smad signaling molecules and inhibits the phosphorylation of TGFβ and Smad2/3. Abnormal expression of Cav-1 may lead to dysregulation of the TGFβ/Smad signaling and alterations of the extracellular matrix (Le Saux et al., 2008). DJ-1/park7 is a multifunctional protein implicated in several biological activities. DJ-1 has been reported to ameliorate hypoxia-induced pulmonary hypertension *in vivo* and *in vitro* by up-regulating Cav-1 expression and inhibiting the TGFβ1/Smad2/3 signaling (Gao et al., 2017).

The influx of Ca^2+^ is mediated by transient receptor potential vanilloid 4 (TRPV4) in ECs. It has been reported that the absence of both TRPV4 and Cav-1 in mice can decrease pulmonary arterial pressure. Cav-1 is colocalized with NOX1 and iNOS, which induce the generation of the oxidant molecule peroxynitrite and promote the pathogenesis of pulmonary hypertension (Daneva et al., 2021). The activation of PPARγ by the specific agonist GW1929 is Cav-1-dependent, and the knockdown of Cav-1 abrogates the upregulation of GW1929 on PPARγ expression. GW1929 significantly decreases the expression of the proliferative mediators and increases the apoptosis-related factors in hypoxia-treated pulmonary arterial smooth muscle cells (PASMCs) (Yang et al., 2018) (Table 1). However, one study shows that the ubiquitin-proteasome inhibitor bortezomib (BTZ) can ameliorate hypoxia-induced pulmonary hypertension by promoting the degradation of Cav-1, which mediates the SOCE/[Ca^2+^]_i_ signaling in PASMCs (Wang et al., 2021).

### 4.5 Pulmonary fibrosis

Idiopathic Pulmonary Fibrosis (IPF), the most common interstitial lung disease, is characterized by progressive lung scarring due to loss of epithelial regeneration induced by chronic lung injury, replicative senescence, and type II alveolar epithelial cell apoptosis. Current anti-fibrotic therapies cannot cure IPF but slow down its progression. The release of pro-fibrotic mediators, the activation of fibroblasts and myofibroblasts, and the secretion of ECM proteins lead to the pathogenesis of IPF (Glassberg, 2019). TGFβ has been reported to be implicated in the fibrogenic process. More specifically, TGFβ stimulates the differentiation of fibroblasts into myofibroblasts and mediates the remodeling of ECM (Vaz de Paula et al., 2021). The expression of Cav-1 is negatively correlated with the TGFβ signaling in pulmonary fibrosis (PF) development (Xing et al., 2017) (Table 1) (Figure 3). Cav-1 has become a therapeutic target for PF treatment, and Cav-1 scaffolding domain peptides (CSPs) have been developed. The full-length CSP (DGIWKASFTTFTVTKYWFYR) can inhibit AEC apoptosis and suppress fibrotic lung fibroblast activation, attenuating bleomycin-induced PF. CSP7 (FTTFTVT), a seven-amino acid fragment of CSP, exhibits anti-fibrotic effects by inhibiting the TGFβ signaling and restoring the expression of p53 (Nagaraja et al., 2018; Marudamuthu et al., 2019). The critical roles of CSP7 in protecting against fibrotic lung diseases have been comprehensively reviewed (Shetty and Idell, 2023).

Excessive collagen content in ECM may result in fibrosis development. Chemoattractant receptor homologous molecule expressed on TH2 cells (CRTH2), a receptor for prostaglandin D2, has been transported to the ER membrane in a Cav-1-dependent manner. CRTH2 deficiency promotes collagen synthesis in fibroblasts and increases the risk of fibrosis (Zuo et al., 2021) (Table 1). Fyn kinase has been implicated in the TGFβ signaling, and Cav-1 is colocalized with Fyn in the caveolae. Dysregulation of the Fyn/TGFβ signaling is involved in the impairment of alveolar barrier function and the development of PF (Menzel et al., 2022). Loss of Cav-1 is associated with reduced alveolar barrier functions and fibrosis-like alterations of the lung parenchyma. Overexpression of Cav-1 protects against PF by suppressing the expression of inflammasome NLRP3 and IL-1β (Lin et al., 2019). The mTOR signaling plays a key role in the pathogenesis of IPF. In *Sftpc-mTOR* ^*SL1+IT*^ transgenic mice, bleomycin treatment promotes severe fibrotic alterations. mTOR activation up-regulates the expression of ANGPTL4, downregulates the expression of Cav-1, and promotes tight junction vulnerability and epithelial-mesenchymal transition (EMT) (Saito et al., 2020). A long non-coding RNA DNM3OS has been verified as a downstream effector of the TGFβ signaling in promoting myofibroblast activation and PF development. DNM3OS can be further processed into three distinct microRNAs, namely, miR-199a-5p, miR-199a-3p, and miR-214-3p. It has been demonstrated that miR-199a-5p promotes PF in a Cav-1-dependent manner (Savary et al., 2019) (Table 1).

Air pollution has been associated with respiratory diseases. It is reported that PM_2.5_ exposure is correlated with the development of PF. Mechanically, PM_2.5_ exposure decreases Cav-1 expression and activates the TGFβ1/Smad2/3, ER stress, and autophagy signaling pathways (Liu et al., 2023). Molybdenum (Mo) and Cadmium (Cd) are harmful heavy metals in the environment. Exposure of Mo and Cd may cause significant pathological alterations, oxidative stress, and iron overload-induced ferroptosis in sheep lungs. Furthermore, Mo- and Cd-activated ferroptosis promotes the pathogenesis of PF by activating the Cav-1/Wnt/β-catenin signaling pathway (Zhang et al., 2023b). CS exposure causes ROS accumulation, interstitial inflammation, and fibroblast proliferation and fibrosis. Combined CS and bleomycin exposure is associated with differential Cav-1 expression, heterogeneous parenchymal remodeling, and an increase in fibrosis (Kulshrestha et al., 2020). In the silicosis mouse models, the expression of Cav-1 is reduced. In Cav-1 knockout mice, wider alveolar septa, increased collagen content, and more silicotic nodules are found. The protective activity of Cav-1 against silica-induced PF might be associated with the suppression of the NF-κB signaling (He et al., 2022) (Table 1). In silica-inhaled mouse models, Cav-1 can attenuate PF by decreasing the expression of YAP1 and suppressing its nuclear translocation for the transcriptional regulation of GLS1 expression (Li et al., 2022).

### 4.6 Lung cancers

Lung cancer is characterized by high malignancy, high morbidity, and high mortality. Early diagnosis and treatment may achieve effectiveness in minimizing the mortality rate of lung cancer. Small cell lung cancer (SCLC) and non-small cell lung cancer (NSCLC) are the two forms of lung cancer. SCLC accounts for approximately 15% of lung cancer cases and is more malignant with a 5 year survival rate of 5%. NSCLC can be divided into squamous cell carcinoma (SCC), adenocarcinoma (AC), and large cell lung cancer (LCLC). It is interesting to find that lung cancer preferentially develops in the vicinity of the fibrotic area in patients with IPF (Kinoshita and Goto, 2019). In addition, the risk of malignancy in patients with IPF is higher up to approximately eightfold than that of general individuals (Jang et al., 2021). A positive correlation of IPF with lung cancer has been demonstrated (Tzouvelekis et al., 2019). However, the underlying mechanisms in mediating the initiation and progression of lung cancers are still unclear. Dysregulation of Cav-1 is associated with cancer progression, such as cell proliferation, migration, apoptosis, and drug resistance. The roles of Cav-1 in lung cancer have been comprehensively reviewed (Blandin Knight et al., 2017). The potential molecular mechanism in protecting against tumor development is related to the interaction of Cav-1 with the molecules in various signaling pathways (Leiser et al., 2021; Yin et al., 2022; Kamposioras et al., 2023).

Cav-1 is a negative mediator of ROS, which is produced by NADPH oxidases (NOX1-5). It has been reported that Cav-1 knockout mice have higher expression of hypoxia-induced NOX-2 and NOX-4 and higher production of superoxide. Treatment of NOX-expression cells with CSP can significantly reverse the effects of hypoxia on human lung microvascular endothelial cells by inhibiting the NF-κB signaling pathway (Chen et al., 2014a) (Table 1) (Figure 3). The antioxidant defense system includes superoxide dismutases (SODs), such as the copper (Cu)/Zn SOD (SOD1) in the cytoplasm, the SOD2 in the mitochondria, and SOD3 outside the cells. SOD3, a Cu-containing enzyme, is produced by vascular smooth muscle cells or fibroblasts and interacts with the endothelial cells. It is reported that Cav-1 deficiency significantly reduces the activity of SOD3, but not SOD1, by promoting the ubiquitination and proteasomal degradation of ATP7A, which is a Cu transporter and colocalized with Cav-1 in the caveolae (Sudhahar et al., 2020). ROS has been reported to facilitate the processes of tumorigenesis under the tumor microenvironment. ROS can disrupt the vessel structure, impair vascular perfusion, and promote the internalization of secreted acidic and cysteine-rich protein (SPARC) protein by enhancing the Tyr14 phosphorylation of Cav-1 (Zhao et al., 2023). Nitric oxide (NO) is frequently increased in tumors and promotes the stability of Oct4, which drives the stemness of lung cancer cells. Mechanically, NO enhances Akt-mediated Tyr14 phosphorylation of Cav-1 and disrupts the interaction of Cav-1 with Oct4, preventing the ubiquitination and proteasomal degradation of Oct4 by Cav-1 in human lung cancer H460 cells (Maiuthed et al., 2018) (Table 1).

MicroRNAs in small extracellular vesicles (sEV-miRNAs) are prepared by tumor cells for communication. A loading pathway of batched tumor-promoting sEV-miRNAs in A549 cells has been designed. Heterogeneous nuclear ribonucleoprotein A1 (hnRNPA1) is selected as a sEV-miRNAs loading protein, and SUMOylated hnRNPA1 in sEVs is used for evaluating the efficacy of sEV-miRNA loading. It is reported that Cav-1 is essential for hnRNPA1-loaded sEV-miRNAs. However, Cav-1 deletion prevents the encapsulation of SUMOylated hnRNPA1 into sEVs and inhibits tumor cell proliferation (Li et al., 2021). LINC81507 is associated with the cell growth, proliferation, migration, and EMT of lung adenocarcinoma by up-regulating Cav-1 expression through sponging miR-199b-5p (Peng et al., 2019). Human serum albumin (HAS) has been identified as a good drug carrier. Cav-1 is reported to promote HAS uptake, developing a biomarker-directed therapy. Recently, an effective HAS-SN-38 conjugate (SSH20) has been developed. Deletion of Cav-1 significantly decreases the uptake and cytotoxicity of SSH20 (Robb et al., 2021) (Table 1).

O-glcNAcylation, a post-translational modification, is associated with metabolic dynamics, cell motility, and protein stability and functions. Cav-1 can be a target of O-glcNAcylation by TRPM7, which is aberrantly expressed in lung cancers. O-glcNAcylation prevents the ubiquitination and proteasomal degradation of Cav-1, leading to the enhanced stability of Cav-1. Inhibition of TRPM7 suppresses cell migration and invasion by mediating the O-glcNAcylation of Cav-1 in NCI-H292 cells (Luanpitpong et al., 2020). PM_2.5_ exposure increases the production of pro-inflammatory cytokines and the EMT and migration of lung cancer cells. Mechanically, PM_2.5_ exposure induces upregulation of PVT1 expression, which sponges miR-199a. Cav-1 is a direct target of miR-199a. Thus, the downregulation of the PVT1/miR-199a/Cav-1 contributes to the inhibition of PM_2.5_ exposure-induced lung cell development (Qi et al., 2021). Cav-1 can form a complex with E-cadherin, which is associated with β-catenin (Sun et al., 2012). However, one study reports that inhibition of Cav-1 enhances the migration and invasion of human lung cancer cell line NCI-H460 by decreasing E-cadherin expression and increasing the Wnt/β-catenin signaling (Song et al., 2012) (Table 1). It has been reported that approximately 40% of NSCLC patients develop brain metastasis, resulting in a dismal prognosis. Enhanced Cav-1 expression after brain metastasis in lung primary tumors has been found. Knockdown of Cav-1 suppresses the migration and invasion of lung cancer cells due to the downregulation of SNAIL expression (Kim Y. J. et al., 2019).

A bioinformatics study using GO, KEGG, and PPI analysis of lung cancer reveals that Cav-1 can be a prognostic predictor for patients with lung cancer. It has been demonstrated that Cav-1 acts as a tumor suppressor, and Cav-1 alterations have been associated with poor prognosis in patients with lung cancer. Cav-1 has been involved in radio-resistance and tumor progression in lung cancer (Leiser et al., 2021). It has been demonstrated that Cav-1 is overexpressed in NSCLCs irradiation (IR)-resistant cell lines H358-IRR and A549-IRR. Cav-1 promotes autophagy and enhances IR-resistant cell survival by increasing the expression of immunity-related GTPase family M protein (IRGM) (Chen et al., 2021) (Table 1). Molecular targeted therapy has advanced the management of various diseases. EGFR-tyrosine kinase inhibitors (TKIs) have been used to treat NSCLC. However, the acquired resistance to EGFR-TKIs limits their clinical applications. It is reported that atorvastatin can effectively suppress tumor growth in EGFR-TKI-resistant NSCLC cells by inhibiting the interaction of Cav-1 with GLUT3, which mediates the uptake of glucose (Ali et al., 2019).

## 5 Clinical perspectives

Human adenovirus type 26 (HAdV26) has been extensively explored for vaccine development. Recently, HAdV26-based vaccines against Ebola and COVID-19 in the European Union have received marketing authorization. The αvβ3 integrin has been reported to be a receptor for HAdV26 in cell infection (Nestić et al., 2019). One study shows that αvβ3 integrin-mediated HAdV26 infection is associated with endocytosis in a Cav-1-dependent manner in A549 cells (Nestić et al., 2022). Aging may increase the susceptibility to infections and decrease vaccine efficacy. It has been reported that the expression of flagellin (FlaB)-dependent TLR5 is not significantly affected by aging in old macrophages. The expression of TLR5 is mediated by Cav-1 through their interaction, and Cav-1 is involved in FlaB-dependent TLR5 signaling. FlaB can be a mucosal adjuvant in aged mice. In *Streptococcus pneumonia*-infected mice, FlaB-PspA fusion exhibits a higher IgG and IgA response and TLR5 enhances the immune responsiveness (Lim et al., 2015).

Mesenchymal stem cell (MSC)-based therapy has received increasing recognition due to the high activities in MSC proliferation and multidirectional differentiation. The Cav-1^F92A^-modified rat BMSCs (rBMSC/Cav-1^F92A^) have been prepared. Treatment with rBMSC/Cav-1^F92A^ can significantly decrease right ventricular systolic pressure, vascular stenosis, and oxidative stress in monocrotaline (MCT)-induced rat pulmonary hypertension. Mechanically, rBMSC/Cav-1^F92A^ suppresses oxidative stress by mediating the CA1/kininogen and SelW/14-3-3*η* signaling pathways via activating the eNOS/NO/cGMP pathway (Yu W. C. et al., 2019). In addition, rBMSC/Cav-1^F92A^ decreases the expression of TNFα, TGFβ1, thrombospondin-1, and MGP and increases SM22α and H-caldesmon expression. Cav-1^F92A^ increases the production of anti-inflammatory cytokines IL-4 and IL-10 and decreases IL-1α and TNFα (Yu W. et al., 2019). Consistently, intravenous delivery of rBMSC expressing eNOS/Cav-1^F92A^ to rats with pulmonary hypertension suppresses PASMC proliferation and improves vascular remodeling by activating the KLF4/p53 signaling pathway (Chen et al., 2017). Another study shows that adipose tissue-derived mesenchymal stem cells (ADMSCs) show inhibitory effects against bleomycin-induced PF by mediating the Cav-1/NF-κB signaling pathway (Chen Z. et al., 2022).

In senescent hMSCs, the expression of Cav-1 is upregulated, and the EGF signaling is attenuated. Overexpression of Cav-1 may lead to inhibition of ERK1/2-mediated insulin signaling and PPARγ-induced adipogenic differentiation in young hMSCs (Park et al., 2005). However, one study shows that the expression of Cav-1 is downregulated in human lung adenocarcinoma. Cav-1 expression is correlated with oncogenic K-Ras-induced premature senescence. Overexpression of Cav-1 can restore cellular senescence in A549 and H460 lung cancer cells (Volonte et al., 2018). In *Cav-1* knockout mice, the osteogenic potential of bone marrow-derived mesenchymal stem cells (BMSCs), bone formation rate, and bone mass have been significantly enhanced. SiRNA-mediated Cav-1 knockdown can enhance the proliferation and osteogenic differentiation of human MSCs (Baker et al., 2012). This differential effect of Cav-1 in stem cells might be associated with the different contexts and microenvironment.

Consistently, bone marrow (BM) transplantation from healthy mice to Cav-1 knockout mice exhibits preventive effects against spontaneous development of PH. However, BM transplantation does not affect pulmonary endothelial remodeling (Asosingh et al., 2017). The phase 1a clinical trial (NCT04233814) of LTI-03/CSP7 for treating IPF by dry powder inhalation has been successfully completed. It has been demonstrated that aerosolized CSP7 dry powder is well tolerated without any obvious impact on respiratory functions. A phase 1b clinical trial testing the efficacy and safety of CSP7 in patients with IPF will begin soon (Shetty and Idell, 2023). Several studies have reported that Cav-1 can be a potential biomarker for therapeutic management and prognosis prediction. However, one study shows that the monoclonal Cav-1 cannot be used to distinguish between malignant pleural mesothelioma (MPM) and pulmonary adenocarcinoma (PA), as shown that 32.35% of MPM and 6.5% of PA are positive for Cav-1 expression (Bozdag et al., 2020).

Penehyclidine hydrochloride (PHC) is a novel anticholinergic drug that exhibits protective activity against LPS-induced ALI by suppressing the p38 MAPK and NF-κB signaling pathways (Shen et al., 2009). The protective effects of PHC against LPS-induced ALI might be associated with the upregulation of Cav-1 expression in J774A.1 cells (Wu et al., 2019). SZ168 (Podoplanin (PDPN) monoclonal antibody) (Heng et al., 2023) and Salidroside (Jingyan et al., 2017) show similar protection against LPS-induced ALI. Glycyrrhizic acid (GA), a bioactive compound from licorice, has been reported to protect against LPS-induced ALI by regulating the expression of angiotensin-converting enzyme 2 (ACE2) and the Cav-1/NF-κB signaling pathway. However, an ACE2 inhibitor MLN-4760 can abrogate the protective effects of GA (Chen Y. et al., 2022). The traditional Chinese medicine Qi-Dong-Huo-Xue-Yin (QDHXY) has been reported to treat ALI by mediating the expression of Cav-1 and protecting against inflammatory responses in LPS-treated mice (Xu et al., 2018). Andrographolide pills (AP) is a labdane diterpene lactone isolated from Andrographis, which is a traditional Chinese medicine. AP consistently protects against LPS-induced pulmonary injury and dysfunction by up-regulating the expression of Cav-1, Src, p47^phox^, and p67^phox^ (Yang et al., 2014). YiPingFeng, a traditional Chinese medicine, has been reported to treat PF by up-regulating Cav-1 expression and inhibiting the TGFβ1/Smad2 signaling pathway (Chen et al., 2024). Quercetin, a natural flavonoid distributed widely in plants, exhibits protective effects against PF by restoring the susceptibility of senescent IPF fibroblasts to apoptosis via up-regulating Cav-1 and FasL expression and inhibiting AKT activation (Hohmann et al., 2019). Chrysotobibenzyl, extracted from *Dendrobium pulchellum*, has been reported to inhibit cell proliferation, migration, invasion, and EMT in H460 and H292 cells by suppressing the expression of Cav-1 (Petpiroon et al., 2019).

## 6 Conclusion

Cav-1, a vital protein for transcytosis, endocytosis, and signal transduction, has been involved in regulating the physiological and pathological processes. The critical roles of Cav-1 in lung diseases, such as pneumonia, asthma, COPD, ALI, pulmonary hypertension, PF, and lung cancer, have been demonstrated. Mechanically, Cav-1 interacts with various pathways, such as MAPK, NF-κB, PI3K/AKT, TGFβ, BMP/Smad, and eNOS/NO signaling, which are associated with the pathogenesis of lung diseases. Targeting caveolae and Cav-1 can be a therapeutic strategy in the mediation of cell signaling pathways and the treatment of lung disease. Owing to the versatile effects of Cav-1 in mediating the signaling pathways, strategies relating to the functional regulation of Cav-1 might be a more effective way to treat lung diseases. It is important to elucidate the underlying mechanisms of Cav-1 in the pathogenesis of lung diseases. Although a Cav-1 scaffolding domain peptide CSP7 has been developed, the structural analysis of Cav-1 should be further deepened. More agents like CSP7 should be developed. Till now, no small molecules as the ligands targeting Cav-1 have been reported.

However, Cav-1 is a challenging protein due to its ubiquitous expression in many organ systems, tissue types, and pathological processes. The interaction of Cav-1 with a variety of signaling molecules is complex, and the biological effects of Cav-1 can be positive or negative. The applications of transgenic or gene-knockout models have been explored for determining the effects of Cav-1. Understanding how one set of gene-profile mediation is controlled over the other remains to be elucidated. The systematic effects of Cav-1 on the physiological and pathological events *in vivo* and *in vitro* need further investigation.

## Figures and Tables

**Table:** 

**ADAMTSs**	A disintegrin and metalloproteinase
**ALI**	Acute lung injury
**ASM**	Airway smooth muscle
**BMPR2**	BMP2 receptor
**BMSCs**	Bone marrow-derived mesenchymal stem cells
**BMPs**	Bone morphogenetic proteins
**CTD**	C-terminal domain
**Cav-1**	Caveolin-1
**CBMs**	Caveolin-binding molecules
**CSD**	Caveolin scaffolding domain
**COPD**	Chronic obstructive pulmonary disease
**CS**	Cigarette smoke
**ES**	Embryonic stem
**ER**	Endoplasmic reticulum
**Enos**	Endothelial NO synthase
**EGFR**	Epidermal growth factor receptor
**EMT**	Epithelial-mesenchymal transition
**ERK1/2**	Extracellular regulated protein kinase1/2
**HO-1**	Heme oxygenase-1
**IPF**	Idiopathic Pulmonary Fibrosis
**I/R**	Ischemic reperfusion
**MMPs**	Matrix metalloproteinases
**MAPK**	Mitogen-activated protein kinase
**NTD**	N-terminal domain
**NF-κB**	Nuclear factor kappa-B
**PKC**	Protein kinase C
**PF**	Pulmonary fibrosis
**Treg**	Regulatory T
**STAT3**	Signal transducer and activator of transcription 3
**SNPs**	Single nucleotide polymorphisms
**SODs**	Superoxide dismutases
**TGFβ1**	Transforming growth factor β1
**TRPV4**	Transient receptor potential vanilloid 4
**VEGF**	Vascular endothelial growth factor
